# Supplementary material for: Nanophotonic Pockels modulators on a silicon nitride platform
Source: Nat Commun. 2018 Aug 27;9:3444. doi: 10.1038/s41467-018-05846-6 (PMC6110768; doi:10.1038/s41467-018-05846-6)
Supplement: Supplementary file 1 — Supplementary Information [file 41467_2018_5846_MOESM1_ESM.pdf]

Supplementary Information:
Nanophotonic Pockels modulators on a silicon nitride platform

Alexander *et al.*

Number of pages: 6

Number of figures: 6

SUPPLEMENTARY INFORMATION

Supplementary Note 1. Additional devices

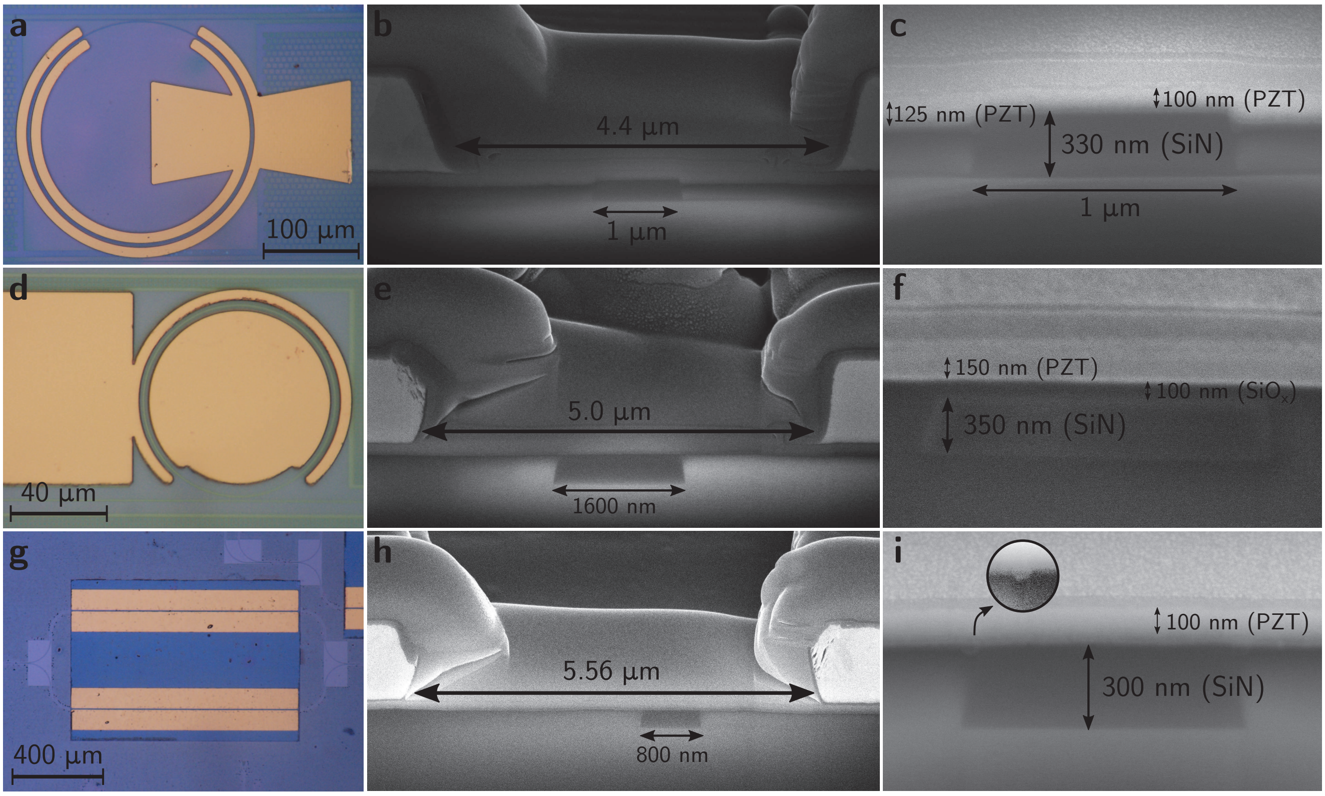

Supplementary Figure 1: Optical microscope and SEM images of the different modulator types. **a**, Top view of a C-band ring modulator. **b**, **c**, Cross-sections of a C-band ring modulator. **d**, Top view of a O-band ring modulator. **e**, **f**, Cross-section of a O-band ring modulator. **g**, Top view of a C-band MZI modulator. **h**, **i**, Cross-section of a C-band MZI modulator, the inset shows the trench at the waveguide edge with enhanced contrast, caused by nonuniform etch rates. The nominal thickness of the intermediate lanthanide layer (below the PZT) is 24 nm in all devices. Nonlinear adjustments were used to enhance contrast in Figs. c, f and i (inset).

The paper mainly highlights the results on C-band ring resonator modulators. However, more device types have been fabricated and characterized. Apart from the C-band rings, this section provides more details on O-band (1260 nm - 1360 nm) ring modulators and a C-band Mach-Zehnder type modulator. Fig. 1 gives an overview: Supplementary Fig. 1a-c show respectively the top-view, cross section and a detailed cross section of the C-band device described in the paper, whereas Supplementary Figs. 1d-f and 1g-i do the same for an O-band ring and a C-band Mach-Zehnder Interferometer (MZI). The O-band ring has a Q-factor of 1820 and a free-spectral range $\Delta\lambda_{\mathrm{FSR}}$ of 3.27 nm. The ring radius is 40 µm, with a phase shifter length L of 195 µm. The MZI modulator electrodes have a length of 1 mm. Note the differences between the cross-sections of the devices, the waveguides in Supplementary Fig. 1c and 1i were planarized trough back-etching of the top ($\approx$ 1 µm thick) oxide using a combination of reactive ion etching (RIE) and wet etching (HF), variations in top oxide thickness, etch rates and exact etch times can lead to relatively large steps (Supplementary Fig. 1c). Moreover, the etch rates of the deposited oxide depend on the exact nitride structures underneath, even in the case of a seemingly planar surface (Supplementary Fig. 1i), trenches of several tens of nanometers arise next to the waveguide (see inset in Supplementary Fig. 1i). On Supplementary Figs. 1d-f however, a device planarized using chemical mechanical polishing (CMP) is shown. A buffer layer of 50-100 nm of oxide is left on top of the nitride waveguide, so the obtained surface is much smoother. This leads to much smaller propagation losses (see Supplementary Note 2).

Supplementary Figs. 2a-c respectively show the transmission spectrum of the O-band ring (pictures in Supplementary Figs. 1d-f), its transmission spectrum for different DC-voltages, and the resonance shift as a function of voltage.

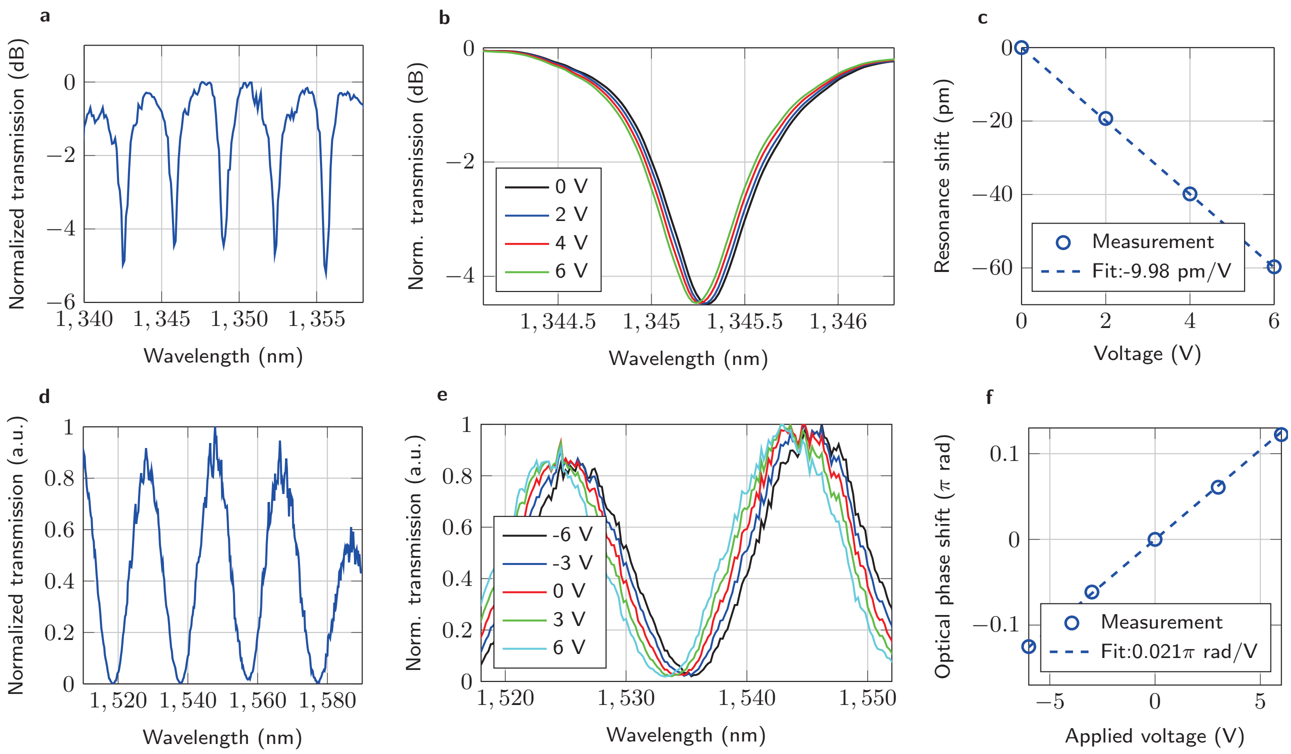

Supplementary Figure 2: Static response of O-band ring and C-band Mach-Zehnder modulators. **a**, Normalized transmission spectrum of the O-band ring. **b**, Transmission spectra for different DC voltages. **c**, Resonance wavelength shift versus voltage, including a linear fit. **d**, Normalized transmission spectrum of the C-band MZI. **e**, Transmission spectra for different DC voltages. **f**, Optical phase shift as a function of voltage, including linear fit.

The linear fit on Supplementary Figs. 2c shows a resonance tuning efficiency of $\Delta\lambda/\Delta V \approx -10$ pm V^{-1}. From this value the half-wave voltage-length product can be estimated: $V_\pi L = |L\lambda_{\mathrm{FSR}}\Delta V/(2\Delta\lambda)| \approx 3.19$ Vcm. Through simulation of the optical mode and DC electric field, the effective electro-optic coefficient r_{eff} of the PZT-layer around 1310 nm is estimated to be about 67 pm V^{-1} (see Methods).

Supplementary Figs. 2d-f show the transmission spectrum of the MZI modulator (pictures in Supplementary Figs. 1g-i), the transmission spectrum for different voltages applied across the PZT and the electro-optic phase shift (with respect to 0 V) as a function of voltage. The voltage is applied to only one of the MZI arms. From this, we can estimate the V_π (voltage needed to induce a π phase shift, or a shift of the sinusoidal transmission pattern over half a period) to be 47.6 V, corresponding to a $V_\pi L$ of 4.76 Vcm. This corresponds to an r_{eff} of the PZT-layer of about 70 pm V^{-1}.

Variations in the measured $V_\pi L$ values are mainly due to variations in the waveguide cross-sections, electrode spacings and the used wavelengths (C-band versus O-band), see Eq. (2). Extracted electro-optic coefficients r_{eff} also vary somewhat, differences can in part be due to variations in film quality on different samples, but mainly stem from small uncertainties on the exact cross-section dimensions.

Supplementary Note 2. Waveguide loss measurements

In Supplementary Fig. 3 the loss measurements on different types of PZT-covered waveguides without metallic contacts are summarized. For the C-band measurements, chips were planarized using a combination of reactive ion etching (RIE) and wet hydrogen fluoride (HF) etching. Typically resulting in steps and trenches of several tens of nanometers in the vicinity of the waveguide (see Supplementary Note 1 and Supplementary Fig. 1c, i). Supplementary Figs. 3a-c summarize loss measurements on such waveguides, for a set of rib waveguide spirals (blue line on Supplementary Fig. 3a and Supplementary Fig. 3b) and a set of wire waveguide spirals (green line on Supplementary Fig. 3a and Supplementary Fig. 3c). The PZT-covered wire waveguides, resembling the ones used in the C-band modulators, have an estimated loss of 5 to 6 dB cm^{-1}. The rib waveguides were defined using a partial etch of 220 nm next to the waveguide core, the influence of this on the propagation loss is only expected to be minor, as is demonstrated by the measurements. Note that before PZT deposition, the waveguide loss can be as low as 0.5 dB cm^{-1}. For the O-band measurements, the planarization of the waveguides was done by chemical-mechanical polishing (CMP), resulting in a waveguide cross-section as shown in Supplementary Fig. 1f, with a residual oxide layer of 50 to 100 nm on top of the waveguide (see Supplementary Note 1). Supplementary Figs. 3d-e show the loss measurements of such waveguides for 3 test samples. The smoother surface for the PZT deposition can result in losses below 1 dB cm^{-1}. The simulated confinement factor in the PZT layer for the C- and O-band waveguides used in the loss measurements are respectively ≈ 0.23 (for both rib and wire waveguides) and ≈ 0.3.

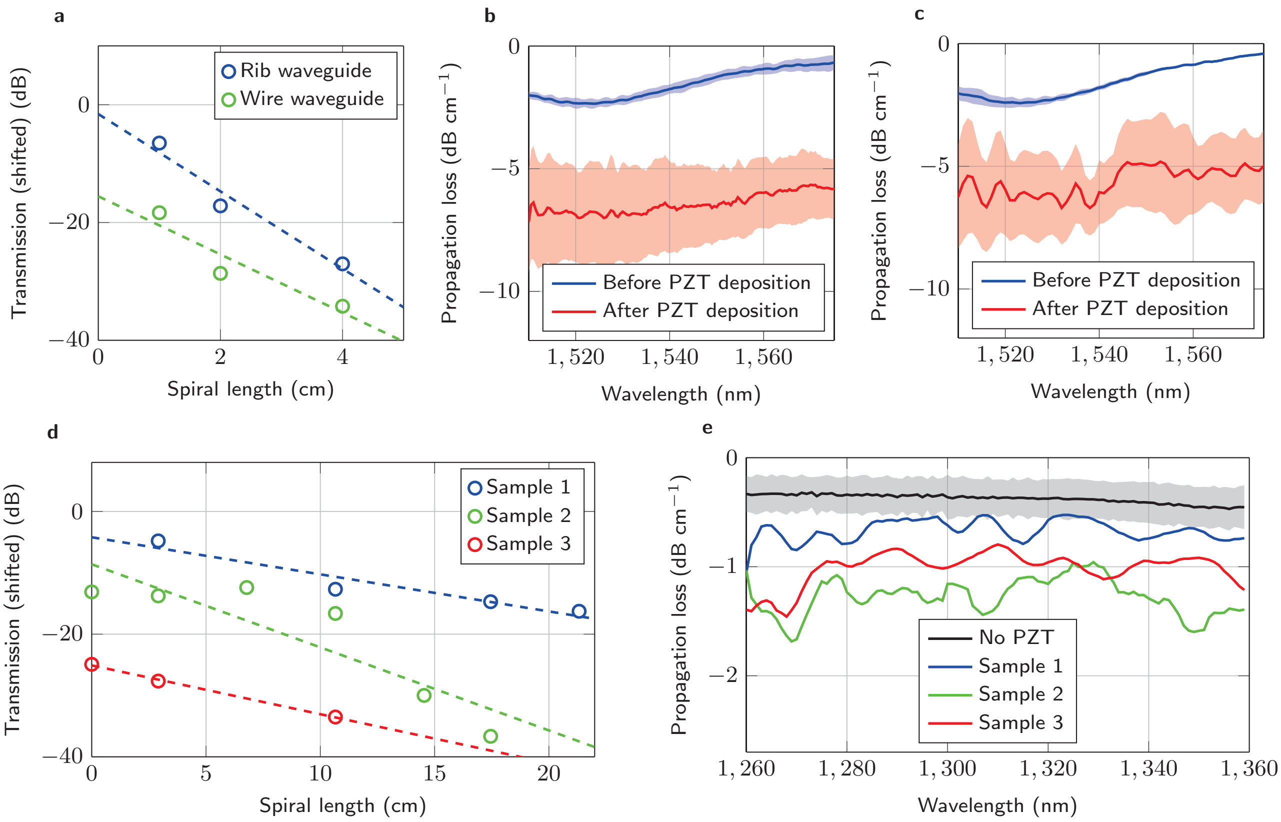

Supplementary Figure 3: Loss measurements. a, Transmission versus waveguide length for a PZT-covered rib and wire waveguide, with cross-section similar to Supplementary Fig. 1c (width = 1400 nm, wavelength = 1550 nm, PZT thickness ≈ 125 nm). **b**, **c**, Propagation loss of the respective rib and wire waveguides versus wavelength, before and after PZT deposition. The shaded area shows the standard deviation on the fitted slope. **d**, Transmission versus waveguide length for a PZT-covered waveguide, with cross-section similar to Supplementary Fig. 1f (width = 800 nm, wavelength = 1310 nm, PZT thickness = 150 nm). Measured on 3 different samples. **e**, Propagation loss versus wavelength for these waveguide sets, including a sample with no PZT.

Supplementary Note 3. Estimation of the bit error rate

Based on the measured eye diagrams, the Q-factors of the eyes and corresponding bit error rates (BER) can be estimated (Ref. [31] of the main text). The results of this analysis are plotted in Supplementary Fig. 4. As can be seen on the plot, the estimated BER remains below the hard-decision forward error coding (HD-FEC) limit with 7% overhead of $3.8 \cdot 10^{-3}$ for all used bitrates (a common limit, see for example Refs. [32, 33] of the main text).

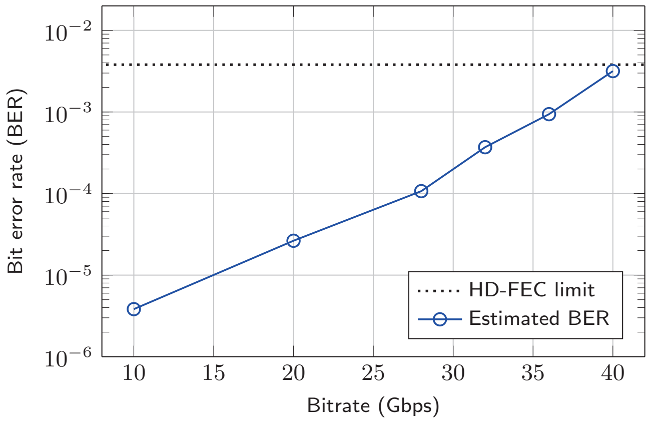

Supplementary Figure 4: BER estimate. Bit error rates estimated on the basis of measured eye diagrams, the horizontal dotted line represents the HD-FEC limit with 7% overhead.

Supplementary Note 4. Extinction ratio measurement

The eye diagram shown in Supplementary Fig. 5 was obtained using a DC-coupled Tectronix 80 C02-CR optical receiver with a sampling oscilloscope (Tektronix CSA 8000), applying a peak-to-peak voltage of 4.2 V at 10 Gbps (same as for Fig. 2c). Since the measured voltage scales with the total optical power, we can estimate the extinction ratio to be about $10 \cdot \log_{10}(P_{\mathrm{max}}/P_{\mathrm{min}})$ dB $\approx \log_{10}(23.8/11.6)$ dB $= 3.12$ dB. This corresponds well with a simple ball-park estimate based on the observed transmission spectrum and static DC-shift (Figs. 1d, e), since the extinction ratio in DC can be estimated as $\Delta T \approx |\left[\frac{dT}{d\lambda}\right]_{\mathrm{max}} \cdot \frac{d\lambda}{dV} \cdot V_{\mathrm{pp}}| \approx 60\ \mathrm{dB\ nm^{-1}} \cdot 13.5\ \mathrm{pm\ V^{-1}} \cdot 4.2\ \mathrm{V} = 3.4$ dB, where T is the transmission expressed in dB.

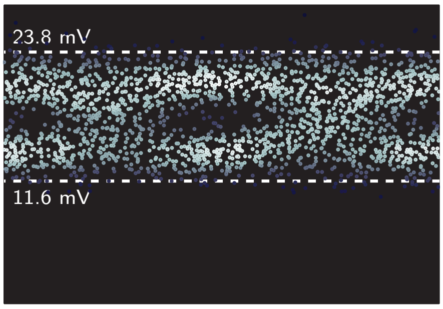

Supplementary Figure 5: Extinction ratio measurement. Eye diagram of a C-band ring modulator, measured with a 10 Gbps non-return-to-zero scheme and a peak-to-peak voltage of 4.2 V (same as in Fig. 2c). Obtained using a DC-coupled optical receiver.

Supplementary Note 5. Device optimization - influence of the waveguide width

In the simulations in Fig. 4, a sweep of the electrode spacing and PZT thickness was performed, since these can be easily tailored in post-processing. This was done for a fixed waveguide width of 1.2 µm. The waveguide width can however also be designed, an optimization is given here. At each width, a sweep of $V_\pi L\alpha$ as a function of PZT thickness and electrode gap of the kind described in the main text and shown in Fig. 4 was performed. Supplementary Fig. 6a shows the optimal (smallest) value $\min(V_\pi L\alpha)$ and the $V_\pi L$ at that optimum. Supplementary Fig. 6b shows the PZT thickness and electrode spacing of this optimum. The light blue area shows the waveguide width/PZT thickness combinations for which the waveguide only supports a single TE mode. In the main text, a width of 1.2 µm was chosen in order to minimize $\min(V_\pi L\alpha)$ whilst still having single-mode behavior at the optimal point.

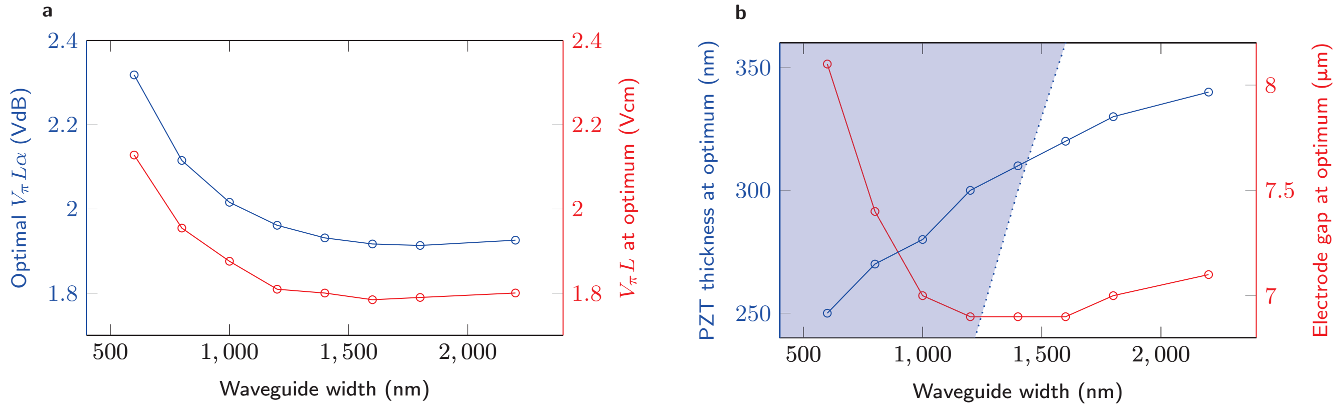

Supplementary Figure 6: Numerical optimization of the a PZT-on-SiN modulator as a function of waveguide width. Optimization of the waveguide loss times the half-wave voltage-length product $V_\pi L\alpha$ of a PZT-covered SiN waveguide modulator as a function of waveguide width. **a**, For each waveguide width $V_\pi L\alpha$ is minimized as function of both electrode spacing and PZT thickness (blue line). The red line plots the calculated $V_\pi L$ at this optimum. **b**, Electrode spacing (blue) and PZT thickness (red) at the optimum. The light blue area shows the waveguide width/PZT thickness combinations for which the waveguide only supports a single TE mode. Wavelength, SiN height and intermediate layer thickness are respectively 1550 nm, 300 nm and 20 nm. The intrinsic waveguide loss (in the absence of electrodes) was taken to be 1 dB cm^{-1}, the effective electro-optic coefficient 67 pm V^{-1}.
